# The short-term prognosis of very late onset generalized myasthenia gravis: a single-center retrospective cohort study

**DOI:** 10.3389/fneur.2025.1641701

**Published:** 2025-08-11

**Authors:** Ioannis Liampas, Dimitra Veltsista, Paraskevi Batzikosta, Lefteris Lazarou, Zinovia Maria Kefalopoulou, Elisabeth Chroni

**Affiliations:** 1 Neuromuscular Center, Department of Neurology, University Hospital of Patras, Patras, Greece; 2 Department of Neurology, University Hospital of Larissa, School of Medicine, University of Thessaly, Larissa, Greece; 3 School of Medicine, Department of Neurology, University of Patras, Patras, Greece

**Keywords:** prognosis, intubation, rescue therapy, relapse, immunosuppressive agents, corticosteroids

## Abstract

**Objective:**

To explore the short-term prognosis of generalized very-late-onset myasthenia gravis (vloMG, symptom onset ≥65 years) in comparison with early- and late-onset MG (eloMG, <65 years).

**Methods:**

A single-center retrospective cohort study was conducted based on the medical records of patients with laboratory confirmed generalized MG, monitored in the specialized Unit of Neuromuscular Disorders of the University Hospital of Patras. Measures of clinical severity were compared at baseline and over the short term (2-year) follow-up.

**Results:**

There were 42 eligible patients (42.1 ± 13.2 years, 50% women, 19.5 ± 6.0 months follow-up) in the eloMG and 26 (72.4 ± 5.0, 50% women, 13.9 ± 7.9 months follow-up) in the vloMG group. In the vloMG group, AchR antibody positivity (89% vs. 57%, *p* = 0.007) and oculo-bulbar symptoms at onset (88% vs. 53%, *p* = 0.002) were more common, whereas thymus pathology (0% vs. 40%, *p* < 0.001) and generalized weakness at onset (12% vs. 38%, *p* = 0.018) were less frequent. Intubation within the first month from diagnosis was required only in patients with vloMG (5/26) (*p* = 0.006). Over the follow-up: the unadjusted incidence rate ratio (IRR) of relapses was lower in the vloMG group [IRR = 0.49, 95% CI = (0.26, 0.92), *p* = 0.026], the unadjusted odds (OR) of being classified as <IIb on the MGFA classification were higher in the vloMG group [OR = 2.27 95% CI = (1.02, 5.05), *p* = 0.043] and the average unadjusted difference in corticosteroid intake was lower in the vloMG group by approximately 6.9 mg [(−10.6, 3.3), *p* < 0.001] in equivalent doses of prednisolone [6.1 mg (−10.0, −2.2), *p* = 0.002, according to adjusted estimations].

**Conclusion:**

Despite its more aggressive onset, vloMG has a more favorable prognosis than eloMG.

## Introduction

Myasthenia gravis (MG) is an autoimmune neuromuscular disorder characterized by muscular weakness with distinctive fluctuation of symptoms and fatiguability. Considerable phenotypic and biological heterogeneity exists, with researchers and clinicians classifying patients with MG by autoantibody profiles and/or the presence of thymic pathology and/or the isolated involvement of extraocular muscles and/or the age of symptom onset (1). Epidemiologic studies report a bimodal incidence with two discrete peaks, one in young adults around the age of 30 years (consistent with the typical high frequency of autoimmune disorders in young women) and one in older adults after the age of 50 years (2). Of note, more recent evidence suggests that the age of MG onset has been significantly altered with an increasingly growing occurrence in individuals older than 65 years (3). Within this group, incidence rates surge at the first half of the 8^th^ decade of life and men are predominantly affected, as opposed to the typical female preponderance of early-onset MG (eoMG) (3, 4).

Based on the age of symptom onset, MG is typically classified as juvenile (onset before 18 years of age) and adult-onset MG (5). The latter group is further divided into early-onset (eoMG, 18–50) and late-onset MG (loMG, >50 years of age) (6). Lately, due to the phenotypic and biological differences of MG occurring above the age of 65, a new MG subgroup is widely recognized; very late-onset MG (vloMG, >65 years of age) (7). In turn, according to the age of onset, adult-onset MG is predominantly categorized as eoMG (18–50 years), loMG (50–65 years) and vloMG (>65 years). Considering their distinctive presentations, the operationalization of these age categories is very important in clinical practice and research. The use of alternative age cut-offs hinders standardized research, conduction of replication studies and data synthesis (8, 9).

Regarding the phenotypic and biological heterogeneity among and between vloMG, loMG and eoMG, it has been reported that ocular MG and male predominance are more frequent in the vloMG and loMG groups (3, 7, 10). The presence of anti-acetylcholine receptor antibodies (AchR Abs) is more common in those with vloMG, whereas thymus hyperplasia and thymoma are less common (3, 7, 10). Of course, the biological differences are anticipated to introduce additional heterogeneity in the clinical course and treatment responses of vloMG in relation to eoMG and loMG (eloMG).

To date, only a few studies have investigated the short-term prognosis of generalized vloMG. Only one focused on the onset of generalized vloMG and found a more severe, potentially more life-threatening onset (7). Several reports support that similar or better clinical outcomes compared to eloMG may be achieved at follow-up, often using lower doses or fewer immunosuppressants (7, 11, 12). On the other hand, conflicting evidence occasionally suggests that individuals with generalized vloMG show worse clinical courses, poorer therapeutic responses and greater dependency on immunosuppressive agents (3, 13). Apart from these inconsistencies, the literature also presents several important methodological drawbacks that limit the value and generalizability of published evidence. The overwhelming majority of published studies performed cross-sectional, unadjusted analyses, occasionally without including an appropriate eloMG group (3, 7, 12, 14–16). Cross-sectional studies do not consider the different monitoring periods of the participants and/or the potential clinical fluctuations over the course of the follow-up. In the case of clinically labile conditions such as MG, it is inappropriate to utilize single (often the last) assessments as prognostic outcomes. Unadjusted analyses fail to account for the well-established biological differences between vloMG and eloMG and capture if a fraction of the variability can be explained by known biological differences. Instead, several researchers have partially addressed this issue by performing subgroup analyses based on immunological profiles and/or thymus pathology; again, this approach may provide some evidence about between group differences but cannot confirm with certainty and quantify these differences. As for studies not involving a proper eloMG comparator, they are not fit to explore the prognosis of vloMG and reveal if vloMG has a relatively more benign prognosis than eloMG.

Therefore, the aim in undertaking the current study was to shed additional light on the short-term prognosis of the generalized vloMG in comparison with eloMG. For this purpose, we conducted a single-center retrospective cohort study. The onset and early 2-year clinical course of generalized vloMG and eloMG were compared. To account for the methodological pitfalls of previous research, our longitudinal investigations took repeated assessments per individual into account. Both unadjusted and adjusted analyses were performed, to determine between group differences and determine if at least part of the variability can be explained by the well-established biological differences between vloMG and eloMG.

## Methods

The present single-center retrospective cohort conforms with the STROBE (Strengthening the Reporting of Observational Studies in Epidemiology) reporting guidelines for observational studies (17). The study protocol was approved by the Ethics Committee of the University of Patras (4,129 /09-02-2023).

### Settings and participants

The current study was based on the medical records of patients with generalized MG who are followed and treated in the specialized Unit of Neuromuscular Disorders of the Neurology Department of the University Hospital of Patras. This Unit was established in 2009 with the aim of monitoring and providing holistic care to patients with neuromuscular disorders, including MG. The current study enrolled individuals followed at the Unit of Neuromuscular Disorders from 2016-onwards. Over the last decade of the Unit’s operation (2016–2025), patients with MG are typically evaluated on approximately 3-month intervals over the first 2 years, after establishing the formal diagnosis. Standardized information is collected on antibody profile, thymus pathology, immunosuppressive agents and dosages, administration of rescue therapies, intubation due to myasthenic crisis, while serial clinical assessments are performed on the quantitative myasthenia gravis scale (QMG) and myasthenia gravis foundation of America clinical classification (MGFA).

### Eligibility criteria

#### Inclusion criteria

- Clinical presentation of generalized fluctuating weakness and fatigability of skeletal muscles over the monitoring period.
- Laboratory confirmed diagnosis of generalized MG. Laboratory confirmation required either positive electrophysiologic investigations (repetitive nerve stimulation or single-fibre electromyography) or positive antibody testing [AchR Abs, muscle-specific kinase antibodies (Musk Abs), low density lipoprotein receptor-related protein 4 antibodies (LRP4 Abs)].
- Initiation of monitoring at the time of diagnosis. Only cases diagnosed or immediately (within 3 months from diagnosis) referred to the Unit of Neuromuscular Disorders and the Neurology Department of the University Hospital of Patras were considered for inclusion.
- Participants’ age at onset ≥18 years.
- Initiation of monitoring from 2016 to date.

#### Exclusion criteria

- Ocular MG with no generalization at any time during the monitoring period.
- Congenital MG.
- Generalized MG following treatment with immune checkpoint inhibitors.
- Indefinite-not laboratory confirmed diagnosis.
- Serious comorbidities impeding the subjective clinical assessments (e.g., quadriplegia due to spinal injury).
- Patients referred to the Unit of Neuromuscular Disorders after being treated at a different medical center for more than 3 months.
- Participants’ age at onset <18 years.
- Initiation of monitoring before 2016.

### Covariates and outcome measures

Data were collected on: age of symptom onset, age of diagnosis, time interval between symptom onset and diagnosis, sex, antibody profile, presence of thymus pathology (thymoma or hyperplasia) and time of surgical removal, predominant symptoms at onset (ocular, bulbar, respiratory, generalized weakness or combination), rescue therapy within 1 month from diagnosis, intubation within 1 month from diagnosis, duration of follow-up, rescue therapies at follow-up (>1 month from diagnosis), QMG scores at baseline and follow-up, MGFA at baseline and follow-up, as well as corticosteroid intake (in equivalent doses of prednisolone, in mg) and rituximab (RTX) or other non-steroidal immunosuppressive therapies (NSITs, azathioprine, mycophenolate mofetil and methotrexate) intake over the follow-up (time of onset and treatment duration).

According to MGFA, participants were dichotomized in two groups: moderate/high MGFA status (MGFA ≥IIb, predominant weakness -even mild- in oropharyngeal and/or respiratory muscles; in case of predominant limb and/or axial symptoms, at least moderate weakness) and mild MGFA status (MGFA <IIb, any ocular weakness ±mild weakness predominantly affecting limb and/or axial muscles) (18). This classification was based on our experience that patients’ expectations are generally satisfied/met when achieving an MGFA <IIb. According to the QMG score, relapses at follow-up were defined as an increase of QMG score by ≥3 from 1 or more items and a total QMG score ≥6 (19). At last (recent) follow-up, the post-intervention status (PIS) achieved was classified as minimal manifestation status or better, improved, unchanged or worse.

### Follow-up

Eligible individuals with generalized MG diagnosed by or referred immediately to (within 3 months from diagnosis) the Unit of Neuromuscular Disorders and the Neurology Department of the University Hospital of Patras were enrolled. The maximum follow-up period for our study was set at 24 months. Therefore, we capitalized on baseline and follow-up visits within the first 2 years of the MG course. Patients with MG are typically evaluated on approximately 3-month intervals, over the first 24 months, in our specialized Unit. Depending on loss at follow-up, the follow-up period ranged between 3 and 24 months per participant. During the follow-up visits, serial clinical assessments involve the QMG (relapses are defined based on this), MGFA categorization and medication intake (dosage of steroid and other non-steroidal immunosuppressive therapies). Unscheduled hospitalizations for rescue therapies are also documented. These data were capitalized on, to compare the short-term prognosis of vloMG and eloMG.

### Statistical analysis

Statistical analyses were performed using the IBM SPSS Statistics Software Version 26 (Chicago, IL, USA). The conventional significance cutoff of *p* < 0.05 was used. Baseline comparisons between vloMG and eloMG were explored using Pearson’s chi-squared test for categorical variables (Fisher’s exact test was used when the expected cell count was below 5) and independent sample *t*-test for continuous variables (Levene’s test was used to assess the equality of variances).

Generalized estimating equations (GEE) were utilized to compare the clinical course of vloMG and eloMG (20). GEE analysis (does not make normality distributional assumptions) takes the multiple visits of an individual into consideration, while accounting for the potential correlation of each participant’ s characteristics over time. Each individual’s repeated measurements were treated as a cluster. Conventionally, exchangeable (compound symmetry) models were chosen as working correlation structures. QMG scores (as linear scale response), MGFA group (as binary logistic response, <IIb or ≥IIb) and counts of (1) rescue therapies and (2) relapses at follow-up (modeled using a negative binomial distribution with a log link) were sequentially inserted as dependent variables in separate GEE models. Both unadjusted (featuring only age group vloMG/eloMG and time) and adjusted (additionally featuring sex, antibody profile, thymus pathology and surgical removal, RXT or other NSIT intake) GEE models were tested. In this way we aimed to capture differences in the clinical course of the two groups and their potential variation due to the well-established biological differences of vloMG and eloMG. Corticosteroid doses were finally inserted into a separate GEE model (as linear scale response), to provide a quantitative measure of treatment differences. Again, both unadjusted and adjusted (featuring the aforementioned set of covariates) GEE models were tested. Finally, PIS was compared between groups using Pearson’s chi-squared test.

## Results

**Figure 1:**
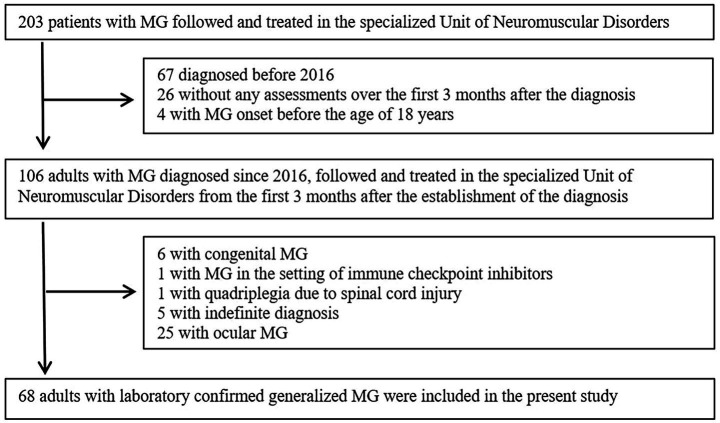
Participants’ flowchart.

From the 203 patients with MG followed and treated in the specialized Unit of Neuromuscular Disorders, a total of 68 adults with laboratory confirmed generalized MG were included in the present study (Figure 1). Among excluded individuals (*n* = 135) based on the exclusion criteria, 80 tested positive for AchR Abs, 9 for Musk Abs, 1 for LRP4 Abs, 24 were seronegative and/or antibody profile was unclear (several diagnoses was established even more than 3 decades before), 6 had congenital MG and 5 had indefinite diagnosis (not laboratory confirmed, with high clinical suspicion – treated as MG).

From the 68 eligible individuals, 42 (42.1 ± 13.2 years, 50% women) were in the eloMG group and 26 (72.4 ± 5.0, 50% women) in the vloMG group. The baseline differences between the two groups are in Table 1. In brief, the time from symptom onset to formal diagnosis was shorter in those with vloMG (*p* = 0.003) and they tested positive for AchR Abs more often than those with eloMG (*p* = 0.007). Thymus pathology was more frequent in the eloMG group (*p* < 0.001), as was generalized weakness at onset (*p* = 0.018). On the other hand, ocular (54% vs. 36%, *p* = 0.142) and bulbar (35% vs. 17%, *p* = 0.090) manifestations at presentation were more common in those with vloMG (*p* = 0.002 for oculo-bulbar symptoms combined). Regarding the early course of the disease, only patients with vloMG (*n* = 5) were intubated within the first month following the formal diagnosis (*p* = 0.006). No difference was revealed with respect to the need for rescue therapies (*p* = 0.393), the average QMG scores (*p* = 0.327) and the proportion of those categorized as <IIb on the MGFA classification, at the time of the diagnosis (*p* = 0.790).

**Table 1:** Participants’ baseline characteristics.

Variable	Total (*n* = 68)	eloMG (*n* = 42)	vloMG (*n* = 26)	*p*-value
Age (in years) at onset	53.7 ± 18.3	42.1 ± 13.2	72.4 ± 5.0	**<0.001**
Time interval (in months) from onset to diagnosis	7.2 ± 10.0	9.6 ± 11.8	3.5 ± 3.5	**0.003**
Follow-up (in months)	17.3 ± 7.3	19.5 ± 6.0	13.9 ± 7.9	**0.004**
Female sex	34 (50%)	21 (50%)	13 (50%)	1.000
AchR Abs (+)	47 (69%)	24 (57%)	23 (89%)	**0.007**
Musk Abs (+)	7 (10%)	6 (14%)	1 (4%)	0.238
LRP4 Abs (+)	2 (3%)	2 (5%)	0 (0%)	0.521
Seronegative MG	12 (18%)	10 (24%)	2 (8%)	0.112
Thymus pathology (thymoma/hyperplasia)	17 (25%)	17 (40%)	0 (0%)	**<0.001**
Ocular symptoms at onset	29 (42%)	15 (36%)	14 (54%)	0.142
Bulbar symptoms at onset	16 (24%)	7 (17%)	9 (35%)	0.090
Generalized weakness at onset	19 (28%)	16 (38%)	3 (12%)	**0.018**
More than one symptom category at onset	5 (6%)	4 (9%)	0 (0%)	0.290
Rescue therapy within 1 month from diagnosis	24 (35%)	12 (29%)	12 (46%)	0.393
Intubation within 1 month from diagnosis	5 (7%)	0 (0%)	5 (19%)	**0.006**
Administration of non-steroidal immunosuppressive treatment during the first 2 years after diagnosis	46 (68%)	32 (76%)	14 (54%)	0.056
QMG at diagnosis	9.8 ± 5.4	9.2 ± 4.8	10.6 ± 6.3	0.327
MGFA ≥IIb at diagnosis	61 (90%)	38 (91%)	23 (89%)	0.790
Relapses at follow-up	0.84 ± 1.0	1.12 ± 1.11	0.38 ± 0.57	**0.001**
Rescue therapies at follow-up	0.44 ± 0.82	0.57 ± 0.94	0.23 ± 0.51	0.058

Categorical data are presented in absolute values (%); continuous data are presented in mean ±standard deviation; *n*, number of participants; MG, myasthenia gravis; eloMG, early and late onset MG group (<65 years); vloMG, very late onset MG group (≥65 years); Abs, antibodies; AchR, acetylcholine receptor; Musk, muscle-specific kinase; LRP4, low density lipoprotein receptor-related protein 4; MGFA, myasthenia gravis foundation of America clinical classification; QMG, quantitative myasthenia gravis score. Bold denotes statistical significance.

**Figure 2:**
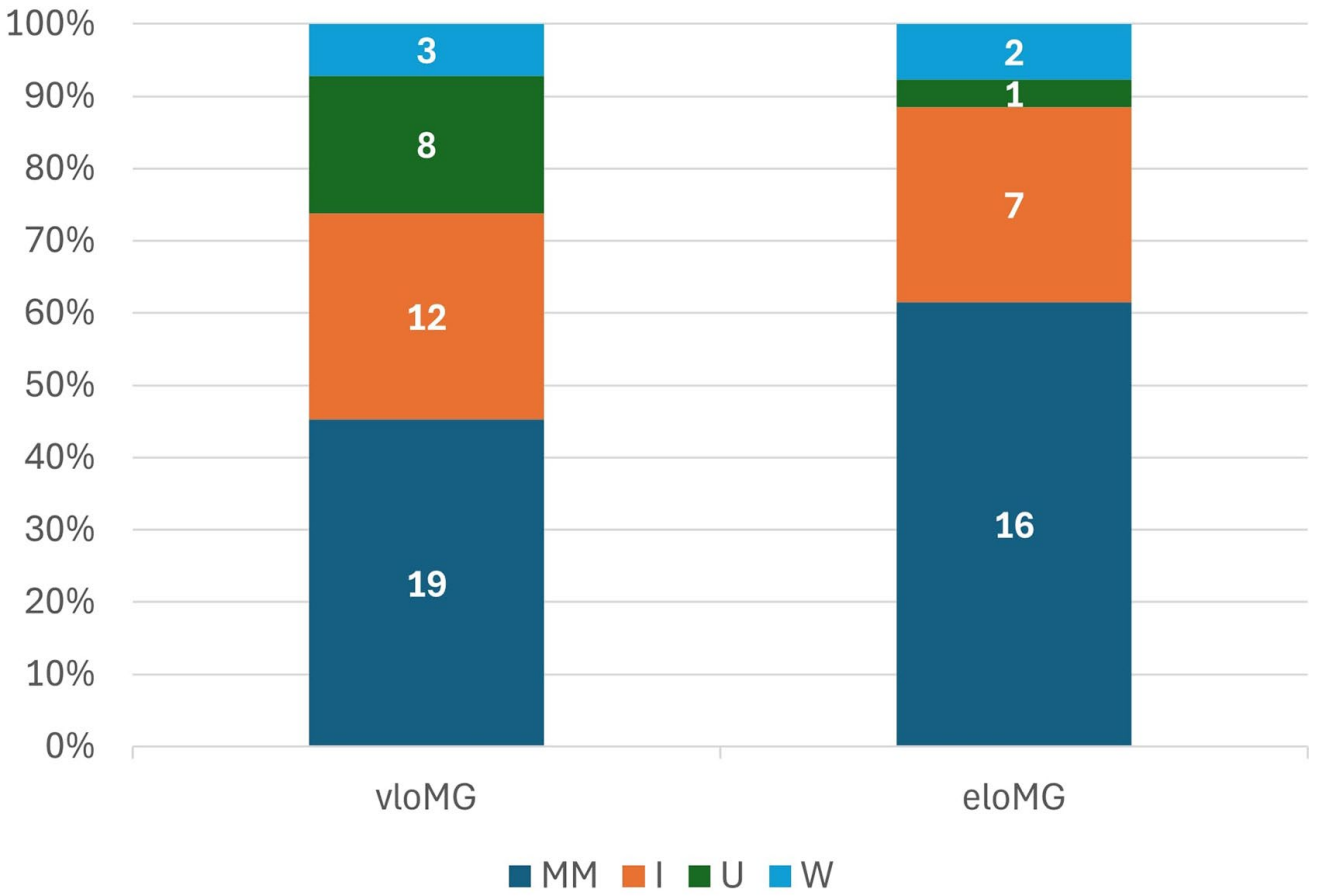
Participants’ distribution by post-intervention status: MM: minimal manifestations or better; I: improved; U: unchanged; W: worse.

Participants were followed for an average of 17.3 ± 7.3 months; those with eloMG for 19.5 ± 6.0 months and those with vloMG for 13.9 ± 7.9 (*p* = 0.004). The results of the longitudinal analyses are in Table 2. The unadjusted incidence rate ratio (IRR) of relapses was lower in the vloMG group, by approximately 50% [IRR = 0.49, 95% CI = (0.26, 0.92), *p* = 0.026]. Moreover, the unadjusted odds of being classified as <IIb on the MGFA classification over the follow-up were more than double in the vloMG compared to the eloMG group [Odds ratio = 2.27 95% CI = (1.02, 5.05), *p* = 0.043]. However, both analyses failed to reach statistical significance after adjusting for the aforementioned set of covariates. Differences in the average QMG scores (about 1 point lower in the vloMG group) and the IRR of rescue therapies over the follow-up (lower in the vloMG group) did not reach statistical significance. Irrespective of failing to reach significant levels, all analyses (adjusted and unadjusted) maintained the same direction of associations (better prognosis in the vloMG group) with lower effect sizes generated after adjusting for important covariates. Finally, PIS was similarly distributed between groups (*p* = 0.301), although a trend toward better outcomes in vloMG was apparent (Figure 2).

**Table 2:** Average longitudinal differences between the eloMG and vloMG groups over the two-year follow-up period.

Outcome	Effect size	95% CI	*p*-value
QMG at follow-up	β1 = −1.08	−2.49, 0.34	0.137
	β2 = −0.87	−2.47, 0.73	0.285
MGFA <IIb at follow-up	OR1 = 2.27	1.02, 5.05	**0.043**
	OR2 = 1.68	0.65, 4.35	0.285
Relapses at follow-up	IRR1 = 0.49	0.26, 0.92	**0.026**
	IRR2 = 0.75	0.39, 1.45	0.399
Rescue therapy at follow-up	IRR1 = 0.59	0.22, 1.58	0.294
	IRR2 = 0.93	0.32, 2.72	0.894
Corticosteroid dosage (in mg)	β1 = −6.9	−10.6, −3.3	**<0.001**
	β2 = −6.1	−10.0, −2.2	**0.002**

The eloMG group was used as reference.

MG, myasthenia gravis; eloMG, early and late onset MG group (<65 years); vloMG, very late onset MG group (≥65 years); *β*, regression coefficient, OR, odds ratio; IRR, incidence rate ratio; CI, confidence interval; MGFA, myasthenia gravis foundation of America clinical classification; QMG, quantitative myasthenia gravis score. 1 stands for unadjusted estimates; 2 stands for adjusted estimates (covariates: time from diagnosis, sex, antibody testing, thymus pathology, thymectomy, azathioprine or mycophenolate or methotrexate intake and rituximab intake). Bold denotes statistical significance.

As for management, the number of individuals that required administration of RTX or other NSITs during the follow-up period was 68%; 76% for the eloMG group and 54% for the vloMG group. This difference came very close to statistical significance (*p* = 0.056). In parallel, the average unadjusted difference in corticosteroid intake over the follow-up was lower by an equivalent of 6.9 mg/d (in equivalent doses of prednisolone) in the vloMG group. A similar (6.1 mg/d), statistically significant difference was estimated after adjusting for important covariates.

## Discussion

We compared the short-term prognosis of vloMG with that of eloMG using a single-center retrospective cohort study design. Our vloMG group featured the same biological characteristics that were previously mentioned, i.e., more frequent AchR Abs positivity, less frequent thymus pathology and an even sex ratio (21). The absence of male preponderance which has been reported by some researchers (3, 12) may be explained by the fact that patients with isolated ocular MG were excluded from our analysis; male sex predominates in ocular MG (22). We demonstrated that vloMG has a more severe, potentially life-threatening onset and a more favorable prognosis, thereafter. Despite the consistent (in terms of direction) though less remarkable (in terms of effect size) adjusted associations, statistical significance was not maintained after accounting for multiple important covariates. This suggests that at least part of the variability of the short-term prognosis is explained by the aforementioned well-established biological differences. However, larger samples are required to better capture these associations and quantify the proportion of the remaining, unexplained variability. By documenting RXT and other NSIT intake and by quantifying average corticosteroid dosage differences over the follow-up, we additionally established that the aforementioned better prognosis was achieved using fewer immunosuppressive agents and lower steroid dosages. The latter was confirmed with both adjusted and unadjusted analyses (similar effect sizes), suggesting that the well-known biological differences between vloMG and eloMG do not play any part in corticosteroid dosage differences.

Age is a crucial risk factor for autoimmunity with many autoimmune disorders occurring after the 5th decade of life and some of them even confined to the elderly (23). This counterintuitive concept of immune aging (considering that immune competence declines with aging) is largely attributed to T cell homeostasis (24). The progressive decline in the regenerative thymic capacity results in an increasing dependence on homeostatic T cell proliferation in adults. This pivotal process in the maintenance of functional T cell populations, may produce autoreactive T cell subsets and amplify autoimmune responses. Despite evidence of increased autoimmunity with aging, many autoimmune disorders usually exhibit milder courses and are less refractory to proper management in older age (25, 26). This age-specific pattern may also be the case in MG (15, 21, 27).

The pattern of a more severe, potential life-threatening onset, but a more favorable prognosis – easier management thereafter in vloMG compared to eloMG disease has been previously reported by some researchers (7). Similar to our sample, ocular muscle weakness is the prevailing initial symptom reported at vloMG onset (3, 21), with the next most common manifestation being bulbar weakness (14, 21). This probably explains, at least partly, the need for respiratory support due to the risk of aspiration. As for the course of MG, most centers treat patients with vloMG with less aggressive immunotherapy regimens. This practice could be attributed to either safety reasons considering possible co-morbidities (3, 14), or to the benign nature of the disease, not requiring high and prolonged doses of immunosuppressive agents (12, 14, 21). In accordance to previous reports, the short-term prognosis in our cohort was more favorable in those with vloMG (7, 11, 12, 28). One possible factor contributing to a better prognosis is the higher number of AChR-positive patients and, respectively, the lower number of seronegative patients in vloMG compared to eloMG. In our experience, seronegativity is more often associated with refractoriness to treatment (29). On the other hand, occasional evidence principally stemming from Asian populations is often contradictory suggesting that older age may be a factor for worse prognosis in MG (3, 13). These differences are most likely attributed to one of the two following reasons. First, considering that older adults often have more numerous and severe comorbidities, sometimes different immunotherapy regimens may be implemented in vloMG. For context, Tang and colleagues reported that poor outcomes in vloMG may be related to the fact that their older cohort included patients who did not receive immunotherapy (3). Second, innate racial, biological differences that have yet to be captured (e.g., genetic polymorphisms that may alter drug metabolism) may explain at least part of the variability in the prognosis of vloMG (30).

Similarly to the majority of relevant studies, our findings are subject to referral and hospitalization biases (31). Although the baseline comparisons of our cohorts have reproduced the well-established biological differences between vloMG and eloMG, our findings do not offer any epidemiological insight. Our sample is rather more representative of more severe cases at the time of formal diagnosis, e.g., cases with severe generalized weakness or bulbar involvement. Regardless, we observed that generalized weakness was the chief earliest complaint in more patients with eloMG than vloMG. Although this finding may be an outcome of the aforementioned referral bias, it could also reflect the increased possibility that generalized weakness may be mistaken for frailty in older adults (32). Therefore, ocular and bulbar symptoms may be more easily associated with MG in older individuals.

The main advantage of the study is that it was conducted in a single center, all patients are monitored by the same team of specialists applying the same practice and individualized choice of medications for older and younger patients. This homogeneity in the management of generalized MG limits the effect of treatment differences on the variability of the short-term prognosis of our cohort. Second, we accounted for the well-established biological differences of vloMG and eloMG and found that at least part of the more favorable prognosis of vloMG should be attributed to these characteristics, i.e., immunological profile and thymus pathology. Previous reports failed to account for these characteristics or performed subgroup instead of adjusted analyses, an approach that cannot quantify between group differences or capture the proportion of variability explained by covariates. Unlike previous cross-sectional studies which failed to capitalize on multiple assessments, we applied GEE analyses, an approach based on the serial assessments per individual, while accounting for the potential correlation of each participant’ s characteristics over time.

The present study has several limitations, as well. Our small sample size has probably underpowered our analyses leading to imprecise estimates, especially in the context of adjusted analyses. However, the consistency of the associations probably reflects that they are non-trivial and larger samples will probably reproduce them (with smaller effect sizes) even after accounting for important covariates. Moreover, although we captured a fraction of the parameters accountable for the variability in the prognosis of vloMG, it is certain that there are numerous other factors that potentially contribute to this variability; comorbidities and medication intake to name a few. Additionally, the retrospective nature of our study may have introduced recall bias. Finally, for the purposes of the current study, we only referred to the medical records kept in our department, which is a center of excellence for neuromuscular diseases. Therefore, a bias toward more severe, treatment refractory cases is anticipated, a factor, however, that is expected to affect both age groups equally.

## Conclusion

We found that vloMG has a more severe, potentially life-threatening onset than eloMG and that oculo-bulbar weakness at presentation raises suspicion of possible MG more often in this age group. The short-term follow-up, however, revealed a more favorable prognosis and a lower dependence on immunosuppressive agents, compared to younger patients.

## Data Availability

The raw data supporting the conclusions of this article will be made available by the authors, without undue reservation.
