# Correction: The short-term prognosis of very late onset generalized myasthenia gravis: a single-center retrospective cohort study

**DOI:** 10.3389/fneur.2025.1716292

**Published:** 2025-11-17

**Authors:** Ioannis Liampas, Dimitra Veltsista, Paraskevi Batzikosta, Lefteris Lazarou, Zinovia Maria Kefalopoulou, Elisabeth Chroni

**Affiliations:** 1 Neuromuscular Center, Department of Neurology, University Hospital of Patras, Patras, Greece; 2 Department of Neurology, University Hospital of Larissa, School of Medicine, University of Thessaly, Larissa, Greece; 3 School of Medicine, Department of Neurology, University of Patras, Patras, Greece

**Keywords:** prognosis, intubation, rescue therapy, relapse, immunosuppressive agents, corticosteroids

Author Dimitra Veltsista was erroneously assigned to affiliation Neuromuscular Center, Department of Neurology, University Hospital of Patras, Patras, Greece. This affiliation has now been removed for author Dimitra Veltsista.

An incorrect Funding statement was provided and displayed as “This research received no specific grant from any funding agency, commercial or not-for-profit sectors”.

The correct funder is Research Council of the University of Patras, the correct funding statement reads:

“The author(s) declare that financial support was received for the research and/or publication of this article. The publication fees of this manuscript have been financed by the Research Council of the University of Patras.”

The original version of this article has been updated.

